# Machine learning enabled subgroup analysis with real-world data to inform clinical trial eligibility criteria design

**DOI:** 10.1038/s41598-023-27856-1

**Published:** 2023-01-12

**Authors:** Jie Xu, Hao Zhang, Hansi Zhang, Jiang Bian, Fei Wang

**Affiliations:** 1 Department of Health Outcomes and Biomedical Informatics, University of Florida, Gainesville, FL USA; 2 Department of Population Health Sciences, Weill Cornell Medicine, New York, NY USA

**Keywords:** Computational models, Data mining

## Abstract

Overly restrictive eligibility criteria for clinical trials may limit the generalizability of the trial results to their target real-world patient populations. We developed a novel machine learning approach using large collections of real-world data (RWD) to better inform clinical trial eligibility criteria design. We extracted patients’ clinical events from electronic health records (EHRs), which include demographics, diagnoses, and drugs, and assumed certain compositions of these clinical events within an individual’s EHRs can determine the subphenotypes—homogeneous clusters of patients, where patients within each subgroup share similar clinical characteristics. We introduced an outcome-guided probabilistic model to identify those subphenotypes, such that the patients within the same subgroup not only share similar clinical characteristics but also at similar risk levels of encountering severe adverse events (SAEs). We evaluated our algorithm on two previously conducted clinical trials with EHRs from the OneFlorida+ Clinical Research Consortium. Our model can clearly identify the patient subgroups who are more likely to suffer or not suffer from SAEs as subphenotypes in a transparent and interpretable way. Our approach identified a set of clinical topics and derived novel patient representations based on them. Each clinical topic represents a certain clinical event composition pattern learned from the patient EHRs. Tested on both trials, patient subgroup (#SAE=0) and patient subgroup (#SAE>0) can be well-separated by k-means clustering using the inferred topics. The inferred topics characterized as likely to align with the patient subgroup (#SAE>0) revealed meaningful combinations of clinical features and can provide data-driven recommendations for refining the exclusion criteria of clinical trials. The proposed supervised topic modeling approach can infer the clinical topics from the subphenotypes with or without SAEs. The potential rules for describing the patient subgroups with SAEs can be further derived to inform the design of clinical trial eligibility criteria.

## Introduction

Appropriately designed clinical studies, especially randomized controlled trials (RCTs), provide gold standard evidence for determining the efficacy and safety of treatments^1^. To maximize internal validity, RCT’s designs usually involve idealized and rigorously controlled conditions with restrictive inclusion and exclusion criteria that define the study population of the trial^2^. Although excessive or overly restrictive eligibility criteria may lower the risk of the study populations for encountering adverse events^3,4^, they usually lead to low population representativeness (thus, low trial generalizability), and subsequently, treatment effectiveness could be reduced, and the likelihood of adverse outcomes could increase when the treatment entered real-world clinical practice^5^. Essential populations of interest are described in Supplement Fig. S1. On the other hand, to be clinically useful, RCT results must be generalizable to the real-world target population in routine clinical practice. External validity, or “generalizability”, is often compromised because of the over-emphasis on internal validity. Low generalizability is a major concern in clinical research communities across disease domains^2,6–8^, including various types of dementias^9–14^ and cancers^15–17^. Therefore, without enrolling the appropriate population, the “true” effectiveness cannot be accurately estimated; and more dangerously, some serious adverse events (SAEs) are not identified until the therapies moved into routine practice, leading to significant patient safety issues and withdrawing drugs from the market^18^.

Regulatory agencies such as the U.S. Food and Drug Administration (FDA)^19,20^ and the broader clinical research communities have called and provided guidance for better trial eligibility criteria (EC) design-e.g., through broadening EC^19^ and using enrichment strategies^20^-to promote enrollment practices so that trial participants can better reflect the real-world target populations and the trials are more likely to succeed. However, trial sponsors and investigators are reluctant to broaden EC concerning about the potential negative impact on the investigational drug’s safety and effectiveness profile. Literature on the concerns of EC design is extensive^2,6–8^, including some of our work^21,22^ However, little effort has focused on providing potentially actionable decision support on choosing the appropriate study population defined by trials’ EC.

Trial generalizability is largely dependent on the representativeness of the study population with respect to the target population to which the study results are intended to be applied^5^. In recent years, the rapid adoption of electronic health record (EHR) systems in the last decade have led to large integrated clinical data warehouses and interoperable clinical data research networks, which made large amounts of real-world clinical data available for research. The National Patient-Centered Clinical Research Network (PCORnet) funded by the Patient-Centered Outcomes Research Institute (PCORI) is one of those examples, that has accumulated data from more than 80 million patients in 2018^23^. These large collections of real-world data (RWD) provide a unique opportunity for studying the impact of EC on (1) the mismatch of the real-world study population and target population they represent, and (2) the consequences of such mismatches in terms of real-world outcomes when the treatment being tested in the trial is applied in clinical practice reflected from the RWD. Insights from these studies can inform and lead to better eligible criteria design of future clinical trials with similar characteristics.

**Figure 1:**
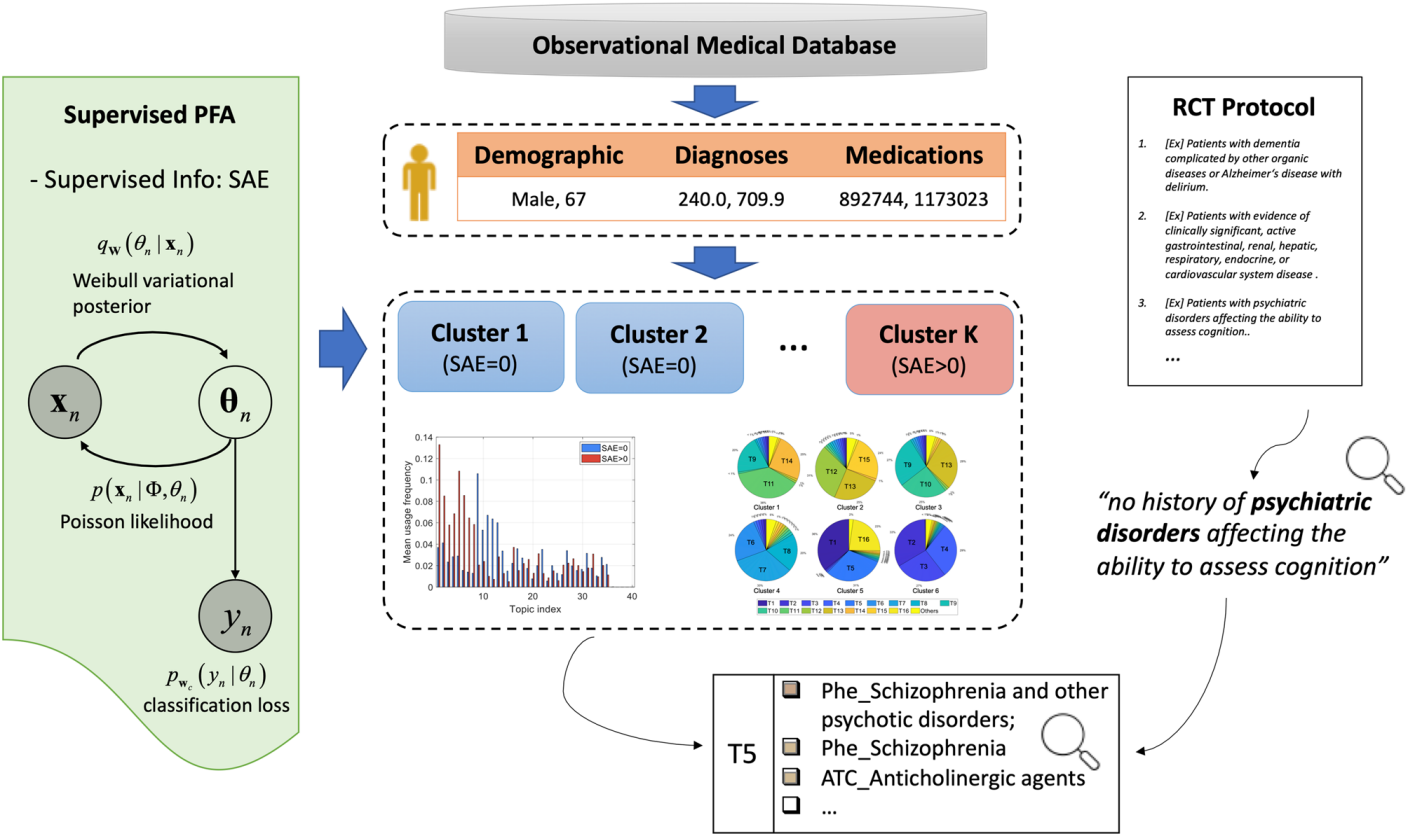
Model overview. Demographics, diagnoses, and medications were extracted from RWD to represent patients. Supervised Poisson factor analysis (PFA) was applied to identify patient subgroups with coherent clinical latent topics and outcomes measured by SAEs. Subgroups with SAEs can be derived to inform the design of clinical trial exclusion criteria.

The goal of this study is to develop machine learning approaches for gaining insights from RWD that could be used to inform clinical trial EC design. In particular, to account for the heterogeneity of the real-world population, we introduce a novel transparent and outcome-guided probabilistic model to identify the subphenotypes (i.e., homogenous clusters of patients) of the target population of a trial (i.e., patients who were placed on the treatment that the trial aimed to develop) (Fig. 1). More importantly, we aim to derive these clusters so that the patients of the target population within the same subphenotype do not just share similar clinical characteristics, but are also predicted to have a similar clinical outcome (i.e., in our current study, we consider patient safety outcome - the risk of experiencing SAEs) after they are placed on the treatment. We hypothesized that certain compositions (i.e., co-occurrence patterns) of the clinical events within an individual’s EHR could determine those subphenotypes and proposed a novel weakly supervised topic modeling approach to identify those subphenotypes, where each clinical topic represents a certain clinical event composition pattern learned from the patient EHRs.

## Methods

### Study design and population

We obtained individual-level patient data from the OneFlorida+ Clinical Research Consortium^24^, which contains robust longitudinal and linked patient-level RWD of ~16.8 million (>60%) Floridians, including data from Medicaid and Medicare claims, cancer registries, vital statistics, and EHRs from its clinical partners. We extracted patients’ clinical care information from OneFlorida+, including patient demographics (i.e., age, sex, race), diagnoses (i.e., coded in International Classification of Diseases 9th/10th revision [ICD-9/10]), and medications (i.e., coded in National Drug Code [NDC] or RXNorm). Uniform-sized bins were used to discretize the age first and then one-hot encoding was adopted to encode the discretized age, gender, and race variables. We mapped diagnosis codes (i.e., ICD-9/10) to Phecode which is designed to facilitate phenome-wide association studies (PheWAS) in EHRs. Drug codes (i.e., NDC or RXNorm) were mapped to the Anatomical Therapeutic Chemical (ATC) Classification System 3rd level. Finally, we concatenated all the features (i.e., demographics, diagnosis, and medications) to represent each patient as a binary vector.

**Figure 2:**
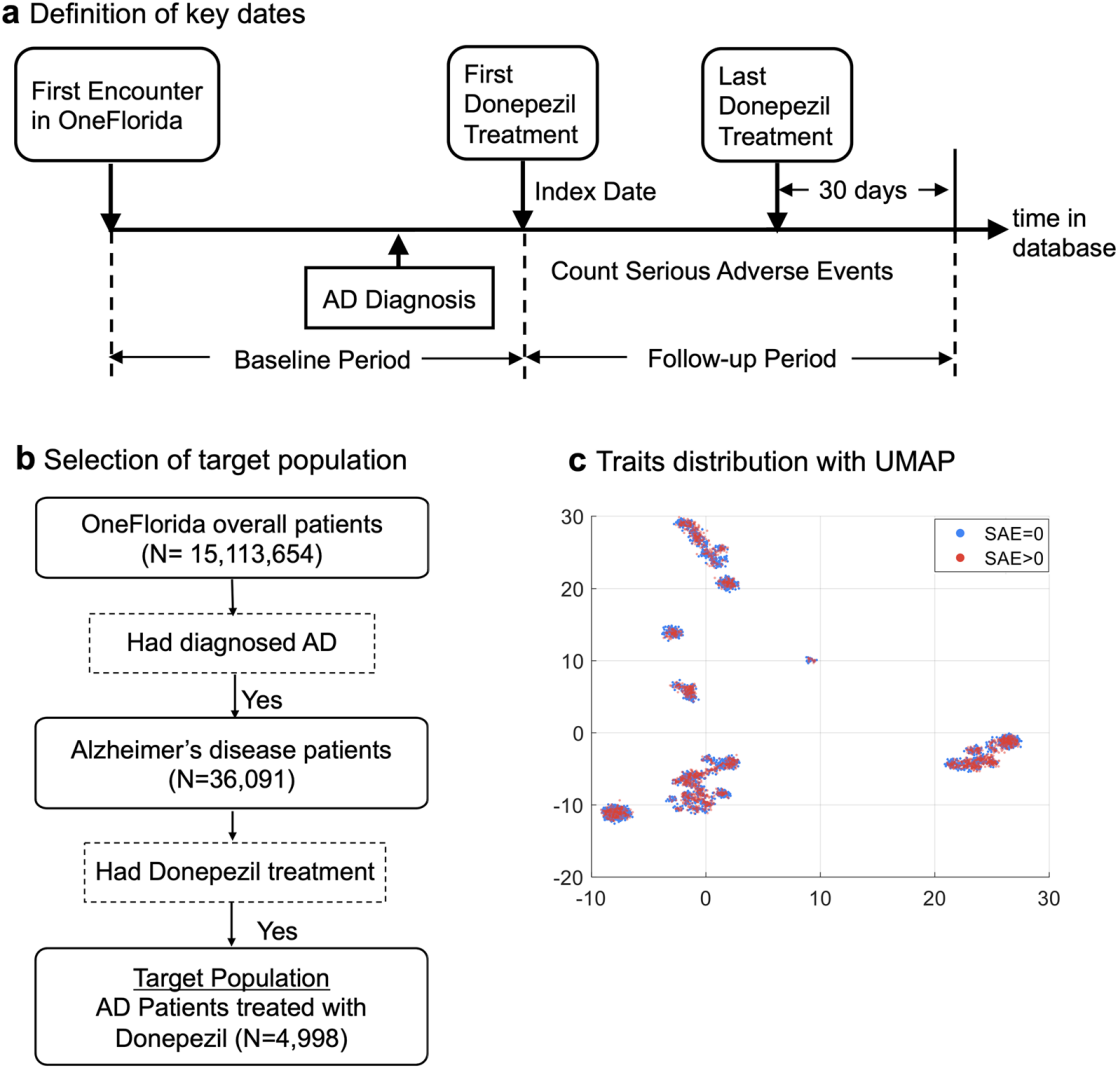
Donepezil clinical trial. (**a**) Definition of key dates. (**b**) Selection of target population. Each sample is colored based on whether the patient had SAEs or not. (**c**) Traits distribution with UMAP.

**Figure 3:**
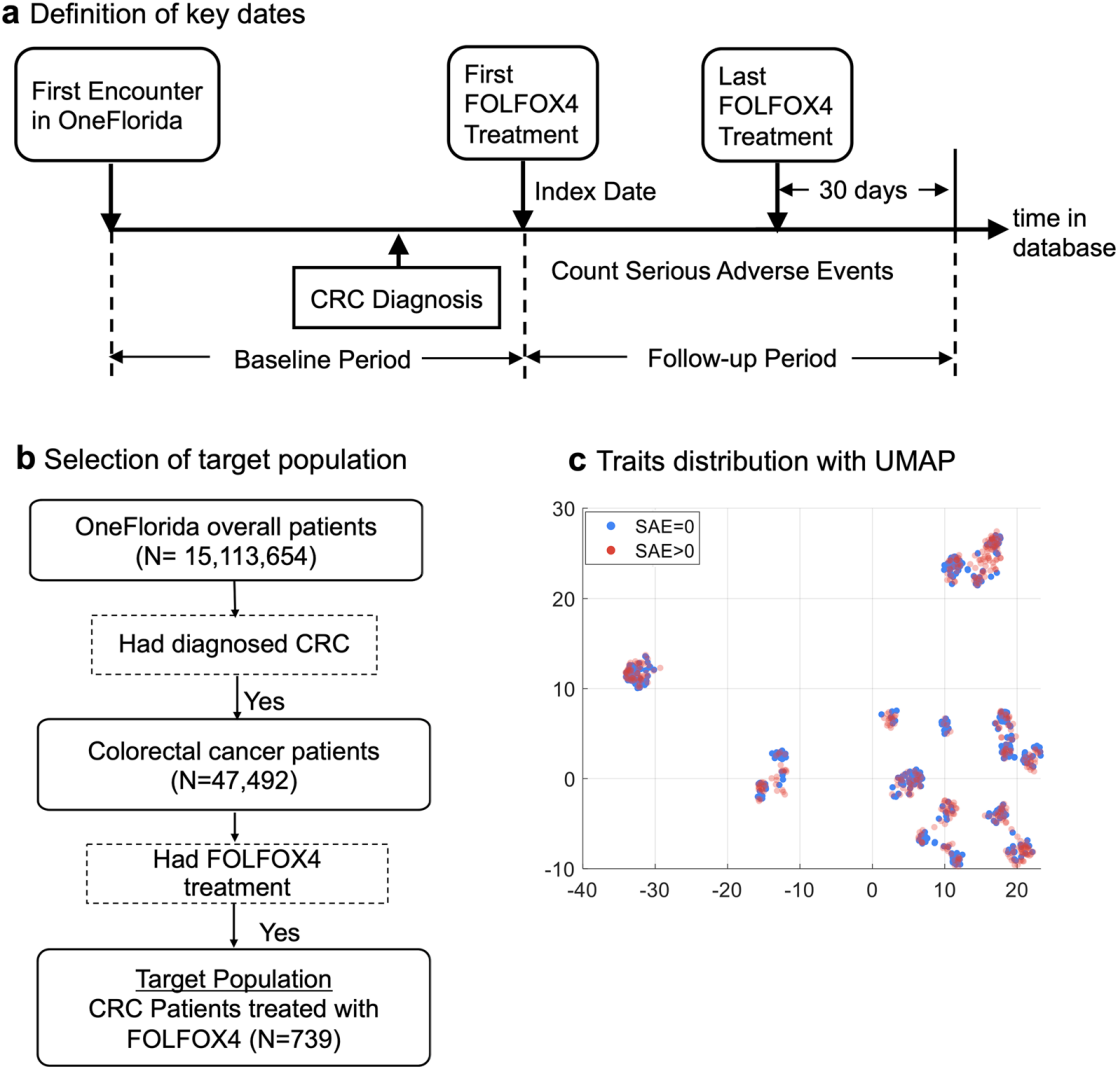
Bevacizumab clinical trial. (**a**) Definition of key dates. (**b**) Selection of target population. Each sample is colored based on whether the patient had SAEs or not. (**c**) Traits distribution with UMAP.

We selected two Phase III RCTs of different disease domains from ClinicalTrials.gov: (1) a hallmark trial (i.e., NCT00478205) that compares the effects of 23 mg to 10 mg donepezil in treating patients with Alzheimer’s disease (AD)^25^; and (2) another RCT (i.e., NCT00112918) studying two different combination chemotherapy regimens with or without bevacizumab (i.e., trade name Avastin) in stage II/III colon cancer patients^26^. For NCT00478205, we set the target population as those who (1) were diagnosed with AD, and (2) were treated with donepezil (Fig. 2b). For NCT00112918, we set the target population as patients who (1) were diagnosed with colorectal cancer (CRC), and (2) were treated with FOLFOX4 (Fig. 3b).

The key dates in our study design are illustrated in Figs. 2a and  3a^21,27^. The beginning of the treatment is set as the index date: (1) the first (ever) observed prescription date of donepezil for NCT00478205, and (2) the first FOLFOX4 treatment after CRC diagnosis for NCT00112918. We refer to the time period before the index date as the baseline period and only use information collected during that time for the clustering analysis. The period from the index date to the last donepezil or FOLFOX4 prescription plus 30 days was set as the follow-up period, from which the SAE information is collected as the patient outcomes.

### Definition of serious adverse events

To define an SAE, we followed two resources: (1) the Food and Drug Administration (FDA)’s definition of SAE^28^, where an adverse event (AE) is considered serious if it results in either death, a life-threatening event, or inpatient hospitalization or prolongation of existing hospitalization; and (2) the Common Terminology Criteria for Adverse Events (CTCAE) - a descriptive terminology for AE reporting^29^, which incorporates certain elements of the MedDRA terminology, e.g., CTCAE terms are grouped by MedDRA primary System Organ Classes (SOCs). Within each SOC, AEs are listed with descriptions of their severity. CTCAE categorizes AE into 5 different severities: Grade 1 (mild), Grade 2 (moderate), Grade 3 (severe or medically significant but not immediately life-threatening), Grade 4 (life-threatening consequences), and Grade 5 (death). To identify SAEs for patients treated with donepezil or FOLFOX4, we first identified the reported SAEs in the Result section of the selected trials from ClinicalTrails.gov, which are organized according to MedDRA. For each SAE, we collected the ICD-9/10-CM codes to identify corresponding health conditions; and we then mapped these health conditions to the CTCAE terms and categorized them as SAEs based on the grading scale above (i.e., CTCAE Grade 3, 4, or 5). Considering both the definitions from FDA and CTCAE, we defined an AE as SAE if it results in hospitalization or death.

### Supervised Poisson factor analysis

By collecting all patient vectors, we can construct a binary data matrix $X\in \{0,1\}^{V\times N}$, with *V* corresponding to the number of features and *N* being the number of patients. Poisson factor analysis (PFA)^30^ assumes *X* following a Poisson likelihood as

1 $$\begin{aligned} X\sim Poisson(\Phi \Theta ) \end{aligned}$$

where $\Phi =[\phi _1,...,\phi _K]\in \mathbb {R}_+^{V\times K}$ is the topic matrix with each column $\phi _k$ being the *k*-th clinical topic, and $\phi _k$ is a distribution over features; $\Theta =[\theta _1,...,\theta _N]\in \mathbb {R}_+^{K\times N}$ is the topic weight matrix and each column $\theta _n$ represents the topic weights of the *n*-th patient. Based on the expectation rule, we have the equation:

2 $$\begin{aligned} x_n=\Phi \theta _n=\phi _1\theta _{n1}+\phi _2\theta _{n2}+...+\phi _K\theta _{nK}. \end{aligned}$$

Clearly, each patient vector is composed of weighted summation of all topics, where values in $\theta _n$ denotes the weights. Therefore, we call $\theta _n$ as the topic weights, a new representation for $x_n$, since it exhibits the weight (or proportion after normalization) of each topic in representing patient $x_n$. We then perform clustering analysis on the learned new representations.

Compared with latent Dirichlet allocation (LDA)^31^, which models the distribution of topic weights as a Dirichlet distribution, PFA models it as a Gamma distribution. The advantage of Gamma distribution for topic weight is that it introduces a shrinkage mechanism to prune inactive factors and enhances the model interpretability^32^. We set the number of topics as 40 for both cases, after learning, our model automatically truncates it to 35 for AD and 26 for CRC. It is in accordance with the fact that our CRC data has less samples, which thus can be described with less topics.

The original PFA is purely unsupervised. In order to incorporate the outcome information (i.e., having SAE or no) into the topic learning process, we extended the original PFA model to a supervised setting which uses the occurrence of SAE as the supervision information to guide the learning process of PFA. Specifically, for the *n*-th patient, if he/she did not encounter any SAE in the follow-up period, we set $y_n=0$; otherwise, we set $y_n=1$. Then we adopted the mean-field variational Bayes method^33^ to maximize the evidence lower bound (ELBO) of the data likelihood as

3 $$\begin{aligned} ELBO=\sum _{n=1}^N\mathbb {E}_{q(\theta _n)}[\log p(x_n|\Phi ,\theta _n)]-\sum _{n=1}^N\mathbb {E}\left[ \frac{q(\theta _n)}{p(\theta _n|r)}\right] , \end{aligned}$$

where $p(x_n|\Phi ,\theta _n)$ and $p(\theta _n|r)$ are the Poisson likelihood and Gamma prior as in PFA, $q(\theta _n)$ is the variational posterior to be learned. Currently, we built $q(\theta _n)$ as an encoder network $q_W(\theta _n|x_n)$, where *W* represents learnable parameters of the encoder network, and $q(\cdot )$ is modeled as a Weibull distribution that makes $\theta _n$ positive and sparse^34^.

To perform supervised learning, we added a supervised regularizer in the original ELBO objective as

4 $$\begin{aligned} L=ELBO+\sum _{n=1}^N\log p_{W_c}(y_n|\theta _n), \end{aligned}$$

where the second term can be viewed as the label likelihood implemented by cross-entropy loss. The model structure of the supervised PFA is shown in Fig. 1. As a result, we built a probabilistic auto-encoding supervised topic model, whose parameters were encoder parameters W, decoder parameters $\Phi$ (topics), and classifier $W_c$. We deployed stochastic gradient descent to learn *W* and $W_c$, and stochastic gradient-based Monte Carlo Markov Chain sampling to infer $\Phi$^34^. Our proposed model can be learned in a mini-batch style, which is easily amenable for large-scale data analysis.

### Clustering with supervised PFA models

Before applying the ML approach to the data matrix, we first represented each patient using the study traits as a vector and checked whether patients with and without SAEs can be well separated. The study traits were extracted corresponding to each computable eligibility criteria and the OneFlorida data. The identified traits included patient demographics (e.g., age) and medical history (e.g., comorbidities and treatments). We dropped the criteria that are not computable (e.g., subjective eligibility criteria such as “written informed consent”).

We then applied supervised PFA (SPFA) and used the occurrence of SAE as the supervision to guide the learning process. Similar to other topic modeling approaches^35^, SPFA first compressed the clinical events into a set of overlapping groups (i.e., topics), and patient representations are derived from these topics based on the idea that groups of clinical events that tend to co-appear in the same visit within the RWD.

K-means clustering is then performed on new patient representations to identify the clusters as subphenotypes. To choose the optimal number of topics, we used all samples to learn the supervised topic model and then evaluated the topic coherence by normalized pointwise mutual information (NPMI) value^36^, and the classification performance by ROC-AUC. We selected the most appropriate number of clusters that provide the largest silhouette score^37,38^.

In our analysis, we used mean topic weight (MTW) to select typical topics. According to the data generation process of PFA and Eq. (1), topic weight of *n*-th patient $\theta _n$ represents the weights of all topics in representing one patient. For fair evaluation, we normalized $\theta _n$ as $\tilde{\theta }_n=\theta _n/\sum _k\theta _{nk}$ to a Dirichelt distribution^31^. As a result, $\tilde{\theta }_n$ can be regarded as topic proportions. Given a group with $\hat{N}$ patients, the MTW of *k*-th topic within this group is calculated as $\sum _{n=1}^{\hat{N}}\tilde{\theta }_{nk}/\hat{N}$. For each topic, after calculation of MTW on SAE subgroup and non-SAE subgroup, we used Mann-Whitney U (MWU) test^39^ to calculate the *p* value of each topic for evaluating the significant difference of topic weights on two subgroups.

### Ethics and dissemination

The study has been approved by University of Florida Institutional Review Board (protocol no. IRB202003137 and IRB202000704). The research has been approved under secondary research for which consent is not required. The research does not involve greater than minimal risk for participation. Analyses only involve the secondary analysis of data that are either limited data sets or de-identified. Our research team has no direct contact with human subjects. All methods were carried out in accordance with relevant guidelines and regulations.

## Results

We report our model results of the donepezil trial (i.e., NCT00478205) and the bevacizumab trial (i.e., NCT00112918) separately below.

### The donepezil trial

A total of 4998 patients (mean (SD) age, 77.53 (9.9) years) were identified from OneFlorida (Table 1). Among which, 3063 (61.3%) had no SAE while 1935 (38.7%) had at least one SAE. Fig. 2c shows the 2D embeddings of patient traits with Uniform Manifold Approximation and Projection (UMAP)^40^. We colored each sample based on whether the patient had SAEs or not. As shown in Fig. 2c, patients with (#SAE>0) versus without (#SAE=0) are intertwined, indicating that the trial-eligible population (i.e., identified by the original trial’s eligibility criteria over our data) in the real world does not guarantee their safety. Further, we examined the differences of the study traits between the two groups (patients with SAE vs. patients without SAEs) through Chi-square tests and summarized the results in Table 1, from which we observe that many traits were not significantly different (statistically, considering $p > 0.05$) including memantine (*p* = 0.145), cancer (*p* = 0.091), antidepressant (*p* = 0.590), basal/squamous cell carcinoma of the skin (*p* = 0.275), galantamine (*p* = 0.190), severe lactose intolerance (*p* = 0.219), and clinically significant Hepatic (*p* = 0.105). There is an opportunity to refine the eligibility criteria that can better predict (thus select) potential participants who are likely to develop SAEs, if it meets the study design goals (e.g., for a safety trial).

**Table 1:** Demographic characteristics and selected traits of the target population of the donepezil clinical trial for AD.

Characteristic	Overall (N = 4998)	# SAEs = 0 (N = 3063)	# SAEs > 0 (N = 1935)	$\chi ^2$ *p* value
Age, Mean (SD), yr	77.53 (9.9)	76.98 (9.8)	78.41 (9.9)	
Sex, No. (%)				
Female	3123 (62.5)	1923 (62.8)	1200 (62.0)	
Race, No. (%)				
White	3,537 (70.8)	2,262 (73.8)	1,275 (65.8)	
Black	965 (19.4)	484 (158)	481 (24.9)	
Asian	34 (0.6)	25 (0.8)	9 (0.5)	
Others & Unknown	462 (9.2)	292 (9.5)	170 (8.8)	
Study traits, No. (%)				
Memantine	1,511 (30.2)	950 (31)	561 (28.9)	0.145
Psychiatric disorders	1,396 (27.9)	754 (24.6)	642 (33.1)	$\le$0.001
Cardiovascular (CS*)	1,082 (21.6)	492 (16)	590 (30.4)	$\le$0.001
Endocrine (CS*)	813 (16.2)	353 (11.5)	460 (23.7)	$\le$0.001
Cancer	808 (16.1)	468 (15.2)	340 (17.5)	0.091
Dysphagia	649 (12.9)	302 (9.8)	347 (17.9)	$\le$0.001
Gastrointestinal (CS*)	631 (12.6)	289 (9.4)	342 (17.6)	$\le$0.001
Drug or alcohol abuse &or dependence	627 (12.5)	283 (9.2)	344 (17.7)	$\le$0.001
Respiratory (CS*)	586 (11.7)	249 (8.1)	337 (17.4)	$\le$0.001
AD with delirium	389 (7.7)	161 (5.2)	228 (11.7)	$\le$0.001
Hepatic disease	361 (7.2)	180 (5.8)	181 (9.3)	$\le$0.001
Renal (CS*)	342 (6.8)	135 (4.4)	207 (10.6)	$\le$0.001
Parkinson disease	329 (6.5)	176 (5.7)	153 (7.9)	0.001
Menopausal	230 (4.6)	128 (4.1)	102 (5.2)	0.040
Antidepressant	226 (4.5)	143 (4.6)	83 (4.2)	0.590
Basal/squamous cell carcinoma of the skin	216 (4.3)	128 (4.1)	88 (4.5)	0.275
Gastric ulcers	163 (3.2)	75 (2.4)	88 (4.5)	$\le$0.001
Inflammatory bowel disease	154 (3)	82 (2.6)	72 (3.7)	0.024
Rivastigmine	153 (3)	121 (3.9)	32 (1.6)	$\le$0.001
Multi-infarct dementia	151 (3)	72 (2.3)	79 (4)	0.001
Acupressure	119 (2.3)	54 (1.7)	65 (3.3)	0.002
Fecal incontinence	107 (2.1)	48 (1.5)	59 (3)	0.001
Galantamine	35 (0.7)	18 (0.5)	17 (0.8)	0.190
Severe lactose intolerance	24 (0.4)	12 (0.3)	12 (0.6)	0.219
Hepatic (CS*)	20 (0.4)	9 (0.2)	11 (0.5)	0.105

CS* Clinically significant. If the disease causes hospitalization, we consider it as “clinically significant”.

**Figure 4:**
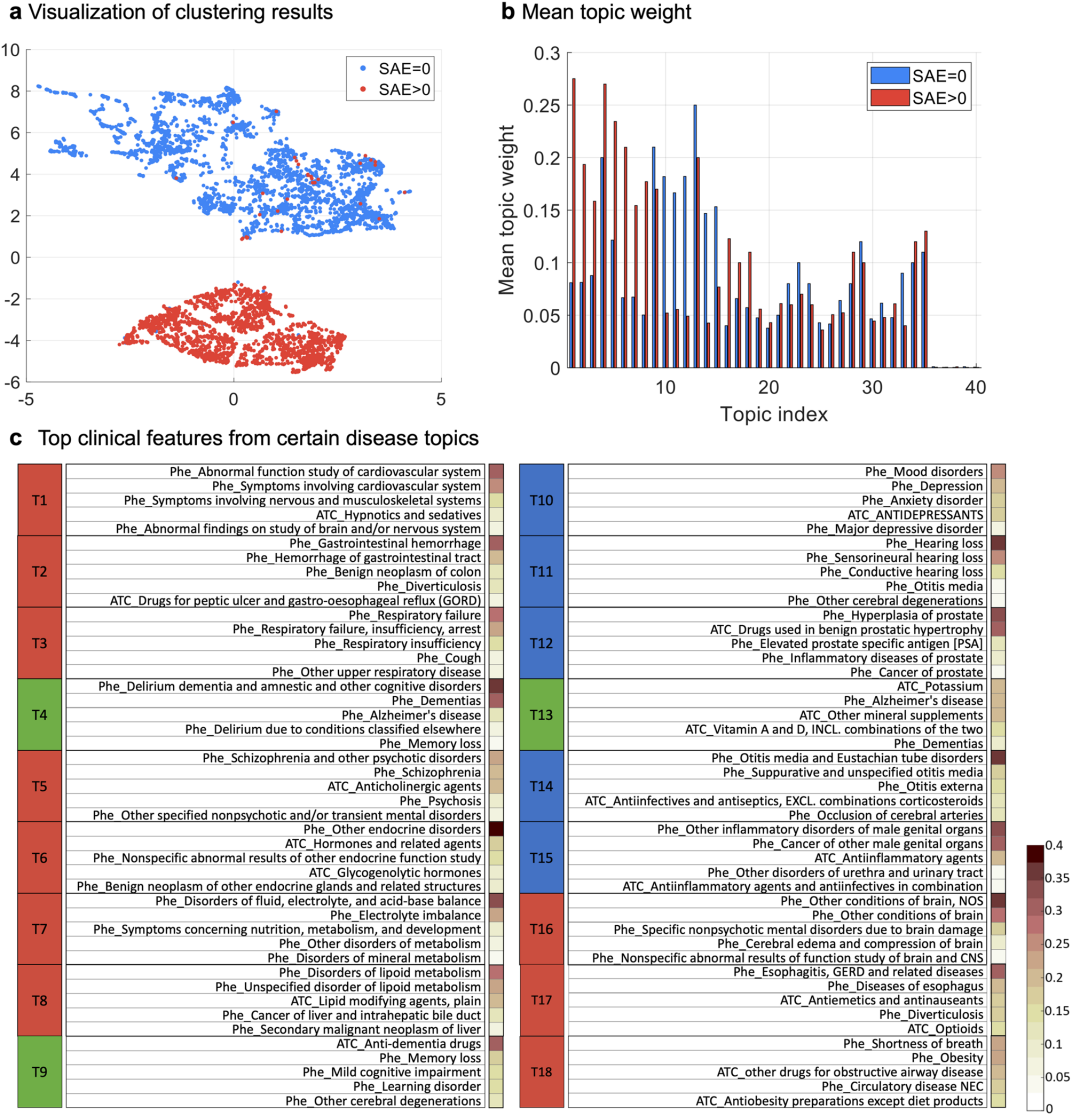
Clustering results of the AD target population. (**a**) Visualization of clustering results. (**b**) Mean topic weight (MTW) of all topics on two groups, where the x-axis is the topic index and the y-axis is the MTW of each topic on two subgroups. (**c**) Top features from certain disease topics. The right sidebar of each topic shows the percentage of patients with the corresponding feature in that topic.

We applied SPFA to the collected data and set #topics=40 for subsequent analyses as it achieved the highest ROC-AUC with large topic coherence values. Six clusters were derived which can be characterized by clinical topics: cluster 1 (N = 1811; 36.23%), patients with disorders of ears or eyes (T11 and T14); cluster 2 (N = 939; 18.79%), patients with diseases of the urinary system (T12 and T15); cluster 3 (N = 331; 6.62%), patients with depression or mood disorder (T10 and T13); cluster 4 (N = 667; 13.35%), patients with disorders of endocrine and metabolism (T6, T7, and T8); cluster 5 (N = 548; 10.96%), patients with different diseases of the brain (T1, T5, and T16); and cluster 6 (N = 702; 14.05%), patients with diseases of digestive and respiratory systems (T2, T3, and T17). Among the six clusters, two patient subgroups emerged: (1) the SAE group (#SAE>0) containing clusters 4, 5, and 6, and (2) the non-SAE group (#SAE=0) including clusters 1, 2, and 3. As shown in Fig. 4a, the two subgroups (i.e., #SAE=0 versus #SAE>0) are well separated, where 1915 out of the 1935 patients (99.0%) in the SAE group encountered SAEs, while 3014 out of the 3063 patients (98.4%) did not have any SAEs in the non-SAE group.

We examined the distribution of the 40 topics across the two subgroups (Fig. 4b). Eighteen topics were then selected for further analysis based on MTW and MWU test^39^. Of the fifteen significantly-different topics (MWU *p*-value$\le$0.05), ten topics (T1~T3, T5~T8, T16~T18, denoted by red in Fig. 4c) were characterized as likely to align with the SAE subgroup and the other five topics (T10~T12, T14~T15, denoted by blue in Fig. 4a) align with the non-SAE subgroup. For the other 3 topics (T4, T9, and T13) whose MWU *p* values$\ge$0.05 but MTWs are in the top three, they are shared by all clusters. We also examined the relevance of the eighteen topics by qualitatively assessing the coherence of the five most prevalent clinical events (i.e., diagnosis and medication codes) for each topic and found that many of the selected topics were specific to different diseases or disease groups (Fig. 4c). Specifically, T4, T9, and T13 include dementia, memory loss, and cognitive impairment-related events, which are shared across the clusters and represent the common diseases and medication use in the cohort. T1 is related to cardiovascular diseases. T2 is related to gastrointestinal diseases. T3 is about respiratory disorders. T5 is related to psychotic disorders, especially Schizophrenia and relevant treatments (anticholinergic agents)^41^. T6 is related to endocrine disorders. T7 is about metabolism disorders such as mineral metabolism disorder. T8 includes lipid metabolism and secondary malignant neoplasm or cancer of the liver, where prior studies have shown the relationship between these two types of diseases^42^. T16 includes various conditions or disorders of the brain, which are closely related to AD. T17 are related to diseases and treatments of the esophagus such as gastroesophageal reflux disease (GERD). T18 is about obesity and some related complications and drugs.

### The bevacizumab trial

A total of 739 patients (mean age, 57.49 with a standard deviation of 11.2 years old) who received FOLFOX4 after diagnosis were identified (out of a total of 47,492 CRC patients) from OneFlorida+. Among all 739 patients, 347 (47.0%) had no SAE, while 392 (53.0%) had at least one SAE. As shown in Fig. 3c, CRC patients with (#SAE>0) vs. without (#SAE=0) are intertwined. We conducted Chi-square tests on the two patient subgroups, i.e., patients who had SAEs (#SAE>0) vs patients who did not (#SAE>0). We found that the *p* values of most study traits are larger than 0.05, except for metastatic disease (*p* = 0.026), parenteral anticoagulants ($p<0.001$), myocardial infarction ($p<0.001$), and thrombolytic agent (*p* = 0.003) as shown in Table 2.

**Table 2:** Demographic characteristics and selected traits of the target population of the bevacizumab clinical trial for CRC.

Characteristic	Overall (N = 739)	# SAEs = 0 (N = 392)	# SAEs > 0 (N = 347)	$\chi ^2$ *p* value
Age, Mean (SD), yr	57.49 (11.2)	59.13 (11.2)	56.0 (11.1)	
Sex, No. (%)				
Female	328 (44.3)	141 (40.6)	187 (47.7)	
Race, No. (%)				
White	488 (66.0)	237 (68.3)	251 (64)	
Black	172 (23.3)	79 (22.8)	93 (23.7)	
Asian	10 (1.4)	5 (1.4)	5 (1.2)	
Others & Unknown	69 (9.3)	26 (7.5)	43 (10.9)	
Study traits, No. (%)				
Colon carcinoma	616 (83.3)	296 (85.3)	320 (81.6)	0.058
Metastatic disease	499 (67.5)	221 (63.6)	278 (70.9)	0.026
Parenteral anticoagulants	240 (32.4)	77 (22.1)	163 (41.5)	$\le$0.001
Immunotherapy	146 (19.7)	75 (21.6)	71 (18.1)	0.130
Anti-angiogenic treatment	137 (18.5)	72 (20.7)	65 (16.5)	0.081
Myocardial infarction	90 (12.1)	25 (7.2)	65 (16.5)	$\le$0.001
Significant traumatic injury	40 (5.4)	16 (4.6)	24 (6.1)	0.379
Thrombolytic agent	38 (5.1)	9 (2.5)	29 (7.3)	0.003
Central nervous disease	37 (5)	12 (3.4)	25 (6.3)	0.099
Inability to take oral medication	33 (4.4)	10 (2.8)	23 (5.8)	0.034
Open biopsy	30 (4)	17 (4.8)	13 (3.3)	0.282
Radiotherapy	24 (3.2)	10 (2.8)	14 (3.5)	0.790
Bone fracture	21 (2.8)	8 (2.3)	13 (3.3)	0.392
Coagulopathy	20 (2.7)	8 (2.3)	12 (3)	0.672
Oophorectomy	17 (2.3)	7 (2)	10 (2.5)	0.956
Cerebrovascular accidents	14 (1.8)	5 (1.4)	9 (2.2)	0.428

**Figure 5:**
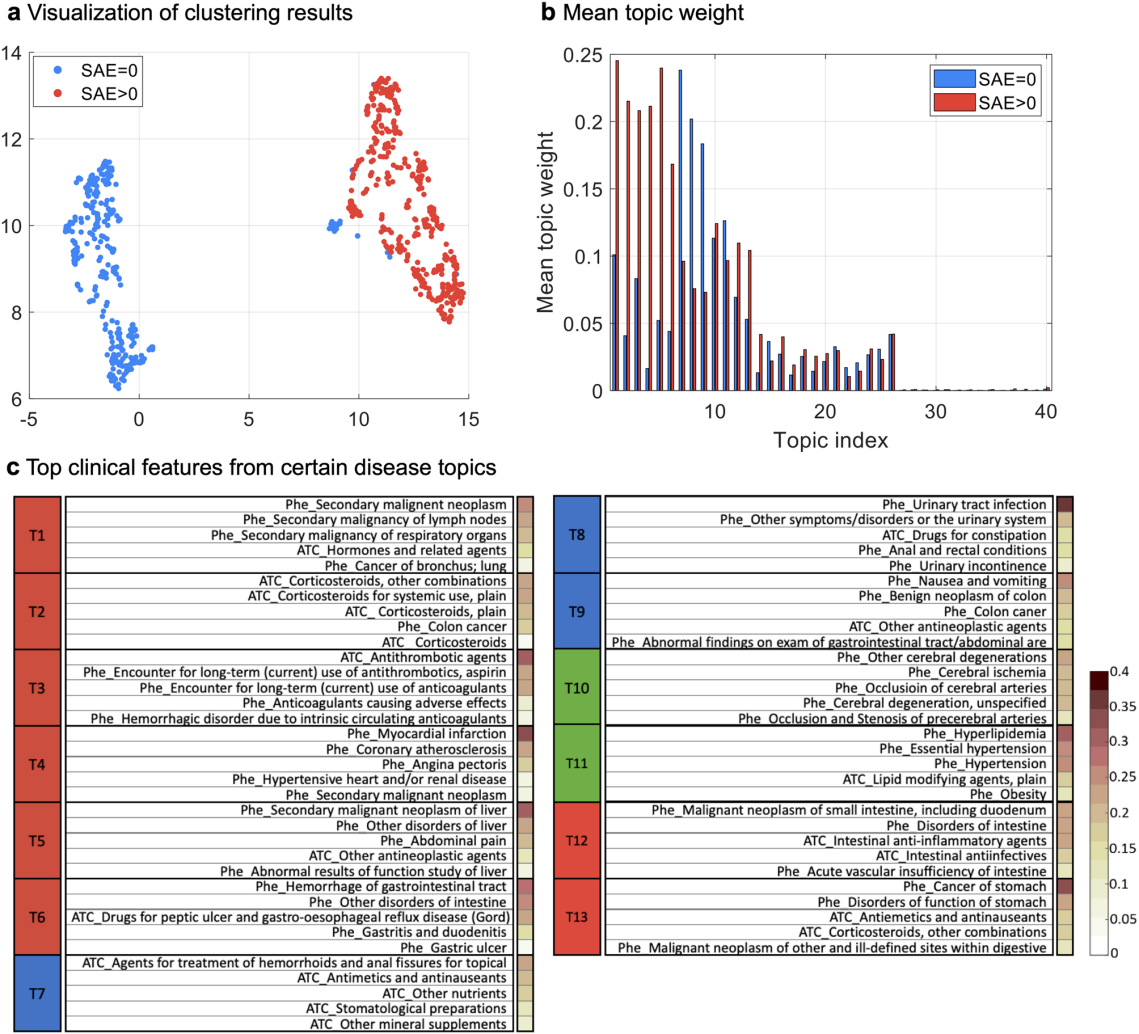
Clustering results of the CRC target population. (**a**) Visualization of clustering results. (**b**) Mean topic weight (MTW) of all topics on two groups, where the x-axis is the topic index and the y-axis is the MTW of each topic on two subgroups. (**c**) Top features from certain disease topics. The right sidebar of each topic shows the percentage of patients with the corresponding feature in that topic.

Similar to the donepezil trial, we applied SPFA to the CRC target population. We set #topics=40 as it achieved higher ROC-AUC and NPMI. Figure 5a shows the UMAP embeddings of new patient representations induced by SPFA, where we can observe two well-separated patient subgroups that can be identified by k-means clustering. One group (red) is mostly associated with patients with SAE, i.e., 317 of 347 patients (91.4%) encountered at least one SAE, and the other group (blue) is free of SAEs (393 patients).

We checked the patient group proportions for the forty learned topics across the two clusters (Fig. 5b). Among all forty topics, using the same topic selection criteria as in donepezil trial, we selected 13 topics for further analysis (Fig. 5c). According to the MTW of the two groups, these topics can be divided into three categories: (1) T1~T6 and T12~T13, represented as red, are associated with the SAE subgroup; (2) T7~T9, represented as blue, aligned with the non-SAE subgroup, contain relatively mild diseases and were not directly related to the diagnosis of colon cancer; (3) T10~T11, represented as green, are often shared on two subgroups. Specifically, T1 is annotated to the use of corticosteroids, with the three of the top five codes being specific corticosteroids treatments. T2 is related to antithrombotic agents. T3 is about malignant neoplasm, where the first three main codes are all correlated with secondary malignant neoplasm and one code is about cancer, and one code is hormones and related preparations drug class which is used to treat cancer. T4 is related to clinically significant (i.e., active) cardiovascular disease. T5 is also related to cancer, but more specifically to the liver. Phenotypes in T6 include various kinds of disorders related to the intestine, e.g., drugs for peptic ulcer and gastro-oesophageal reflux disease (GORD), hemorrhage of the gastrointestinal tract, gastritis, and duodenitis, and gastric ulcer. T7 includes some commonly used drugs. T8 talks about the disease and treatments of urinary tract infection, which is extremely common in the elderly. T9 is associated with gastrointestinal diseases such as nausea and vomiting. T10 and T11 are some common diseases such as or hyperlipidemia or hypertension. T12 includes different diseases or treatments for the intestine.

## Discussion

Rigorous eligibility criteria for RCTs may make the trial participants not representative of the trial’s real-world target patients, where the trial results intended to be applied when the treatment is moved into clinical practice. The FDA, funding agencies, and various research communities have called to broaden eligibility criteria to make clinical trials more representative^15^. Nevertheless, trial investigators and sponsors are hesitant to do so because of their concerns about whether broadening the eligibility criteria would compromise the efficacy results and/or patient safety profiles. There lack of methods and tools to provide such decision support based on real-world data, e.g., so that trial investigators can relax certain eligibility criteria that would not lead to more SAEs.

In this paper, we developed a machine learning approach to identify patient subgroups (i.e., subphenotypes) using large collections of RWD from the OneFlorida+ network that are either more or less likely to encounter SAEs after using the treatment. We consider patient demographics and all clinical events, including diagnosis and medications, in the baseline period for deriving the subgroups. To account for the high dimensionality of RWD, we proposed a novel supervised topic modeling approach that uses the SAE information as a weak supervision. Our approach can effectively identify a set of clinical topics and derived novel patient representations based on them in a lower dimensionality (i.e., from thousands of clinical features to 40 topics), such that the patient subgroups with or without SAEs can be well separated with these representations.

We applied our method using two RCTs from different disease domains: (1) NCT00478205 for AD; and (2) NCT00112918 for CRC. Tested on both trials, patient subgroup (#SAE=0) and patient subgroup (#SAE>0) can be well-separated by k-means clustering using the inferred topics. The inferred topics characterized as likely to align with the patient subgroup (#SAE>0) revealed meaningful combinations of clinical features and can provide data-driven recommendations for refining the eligibility criteria of clinical trials. We analyzed the association between the inferred topics with the SAE subgroup and the extracted computable eligibility criteria. We found that topics aligned with the SAE patient subgroup (#SAE>0) are highly associated with the exclusion criteria of the trial (Tables 3 and 4).

However, compared to the eligibility criteria of the trial, the learned clinical topics provided more detailed information, which prompted us to relax the ambiguous exclusion criteria while making them easier to interpret and implement. For example, for the Donepezil trial, T5 is a combination of schizophrenia and other psychotic disorders and does not mention sleep disorders. So we can relax the corresponding exclusion criterion to “*Patients with schizophrenia and other psychotic disorders.*”. T7 is about disorders of lipoid metabolism, so the corresponding criterion can be relaxed to “*Patients with disorders of lipoid metabolism.*” Even for one disease that appears in both SAE-associated topics and exclusion criteria of the trial, the identified topics provide more detailed insights. For example, for gastrointestinal disease, the exclusion criteria only said “*Patients with evidence of clinically significant active gastrointestinal disease*”, which is a relatively coarse description. However, the learned topics, T2 and T17, discover more detailed diseases or drugs related to gastrointestinal disease. For the Bevacizumab trial, the eligibility criterion provides a rough description of corticosteroids as “*Current or recent (within 10 days prior to study treatment start) use of full-dose oral or parenteral anticoagulants or thrombolytic agents for therapeutic purposes*”. But the topic T2 contains more detailed drugs about corticosteroids. In addition, most topics associated with the non-SAE subgroup are mild comorbidities that are common and may have a lower probability of causing SAE. Therefore, these advantages allow our method to better separate the two subgroups and relax the eligibility criteria.

**Table 3:** Inferred topics and related exclusion criteria in the original donepezil trial (i.e., NCT00478205).

Topics	Related exclusion criteria
T1 (cardiovascular), T2 (gastrointestinal), T3 (respiratory), T6 (endocrine), T8 (lipoid metabolism)	Patients with evidence of clinically significant, active gastrointestinal, renal, hepatic, respiratory, endocrine, or cardiovascular system disease (including history of life-threatening arrhythmias).
T4 (delirium)	Patients with dementia complicated by other organic diseases or Alzheimer’s disease with delirium.
T5 (psychotic)	Patients with psychiatric disorders affecting the ability to assess cognition such as schizophrenia, bipolar or unipolar depression. Patients with clinically significant sleep disorders will also be excluded unless these are controlled by treatment and clinically stable for > 3 months prior to screening.
T7 (metabolism)	Patients with any conditions affecting absorption, distribution, or metabolism of the study medication (e.g., inflammatory bowel disease, gastric or duodenal ulcers, hepatic disease, or severe lactose intolerance).
T8 (lipoid metabolism, secondary malignant neoplasm or cancer of the liver)	Patients with a history of cancer (does not include basal or squamous cell carcinoma of the skin) treated within 5 years prior to study entry, or current evidence of malignant neoplasm, recurrent, metastatic disease. Males with localized prostate cancer requiring no treatment would not be excluded.

**Table 4:** Inferred topics and related exclusion criteria in the original bevacizumab trial (i.e., NCT00112918).

Topics	Related exclusion criteria
T1 (corticosteroids)	Chronic treatment with corticosteroids (dose of $\ge$ 10 mg/day methylprednisolone equivalent) (excluding inhaled steroids).
T2 (antithrombotic)	Current or recent (within 10 days prior to study treatment start) use of full-dose oral or parenteral anticoagulants or thrombolytic agents for therapeutic purposes.
T3 (malignant neoplasm), T5 (cancer, mainly liver related)	Macroscopic or microscopic evidence of remaining tumour. Patients should never have had any evidence of metastatic disease (including presence of tumour cells in the ascites). The isolated finding of cytokeratin positive cells in bone marrow is not considered evidence of metastatic disease for purposes of this study. Other malignancies within the last 5 years (other than curatively treated basal cell carcinoma of the skin and/or in situ carcinoma of the cervix). Previous anti-angiogenic treatment for any malignancy; cytotoxic chemotherapy, radiotherapy or immunotherapy for colon cancer.
T4 (cardiovascular)	Clinically significant (i.e., active) cardiovascular disease. This includes, but is not limited to, the following examples: cerebrovascular accidents ($\le$ 6 months prior to randomization), myocardial infarction ($\le$ 1 year prior to randomization).
T6 (intestine)	Lack of physical integrity of the upper gastro-intestinal tract, malabsorption syndrome, or inability to take oral medication.

In a recent study, Liu et al. evaluated EC for oncology trials using RWD and AI, the authors quantified the representability of each study trait with SHAP, and they tried to relax the range of each eligibility criterion for broadening the participation^43,44^. Only traits with continuous values are considered in a one-by-one manner. Our proposed approach mainly considered binary traits (continuous traits can also be incorporated with appropriate discretizations followed by one-hot representations) and modeled the high-order interactions of these traits as clinical topics. In addition, we also considered adding extra traits to improve the representability and safety of the trial in RWD.

Our study has several limitations. First, our study only leveraged the RWD from OneFlorida, which is a regional clinical research network. Future investigation on larger and more diverse RWD is needed to enhance the generalizability of the identified subgroups. Second, we only explored structured information in RWD in this study. Much of important information, such as symptoms, clinical assessments (e.g., from radiology and pathology reports), and socioeconomic status, are only encoded in clinical notes. Extracting and incorporating unstructured information in our study is another important direction to pursue. Third, only discrete traits have been considered in this study. Continuous traits, such as lab tests, are also crucial for many RCTs. Their corresponding computable counterparts in RWD should be explored as well. Fourth, there are different strategies for “enrichment” (that affect EC design) as recommended by the FDA, for example, “excluding patients unlikely to tolerate the drug” to decrease the nondrug-related variability or “identifying people at relatively high risk” for safety studies^20^. Our study only considered patient safety (i.e., SAEs), while other enrichment strategies that consider treatment effectiveness should also be developed. Nevertheless, our general framework holds the potential to derive insights from RWD that can inform clinical trial design and develop efficient enrichment strategies.

## Supplementary Information

Supplementary Information.

## Data Availability

All data required to evaluate the conclusions of the manuscript are presented in the main text and/or the Supplementary Materials. The dataset used during the current study is a HIPAA limited data set, which requires a data use agreement with the OneFlorida+ clinical research consortium, https://onefloridaconsortium.org/. Request of the data can be sent to the OneFlorida+.
