# Supplementary material for: Machine learning enabled subgroup analysis with real-world data to inform clinical trial eligibility criteria design
Source: Sci Rep. 2023 Jan 12;13:613. doi: 10.1038/s41598-023-27856-1 (PMC9837131; doi:10.1038/s41598-023-27856-1)
Supplement: Supplementary file 1 — Supplementary Information. [file 41598_2023_27856_MOESM1_ESM.docx]

**Supplementary Information: Machine Learning Enabled Subgroup Analysis with Real-world Data to Inform Clinical Trial Eligibility Criteria Design**

**Jie Xu**^1,2^*, **Hao Zhang**^2^*, **Hansi Zhang**^1^, **Jiang Bian**^1#^, **Fei Wang**^2#^

^1^Department of Health Outcomes and Biomedical Informatics, College of Medicine, University of Florida, Gainesville, FL, USA

^2^Department of Population Health Sciences, Weill Cornell Medicine, New York, NY, USA

^*^Equal Contribution.

^#^Corresponding Authors.


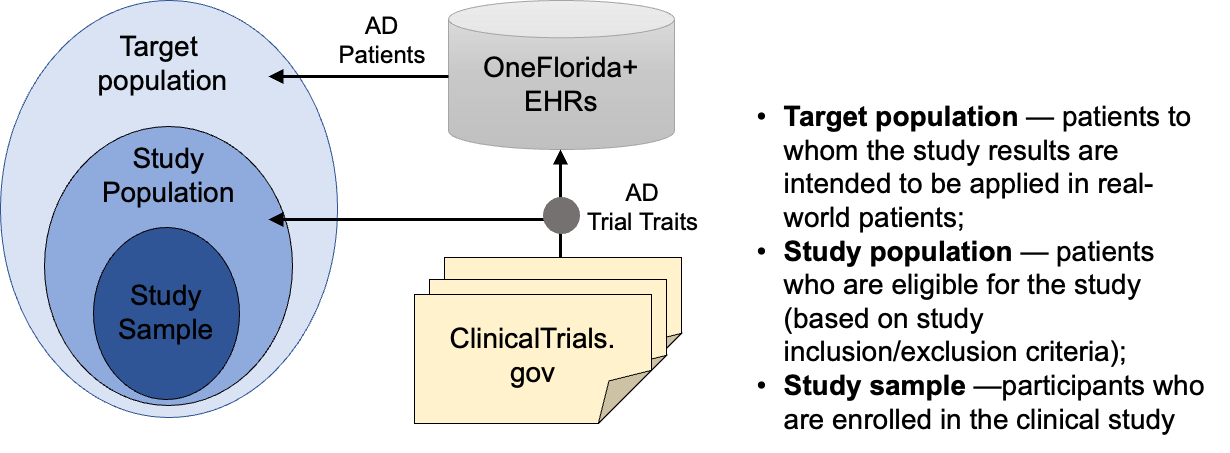


**Figure S1. Illustration of essential populations of interest in clinical trial.**


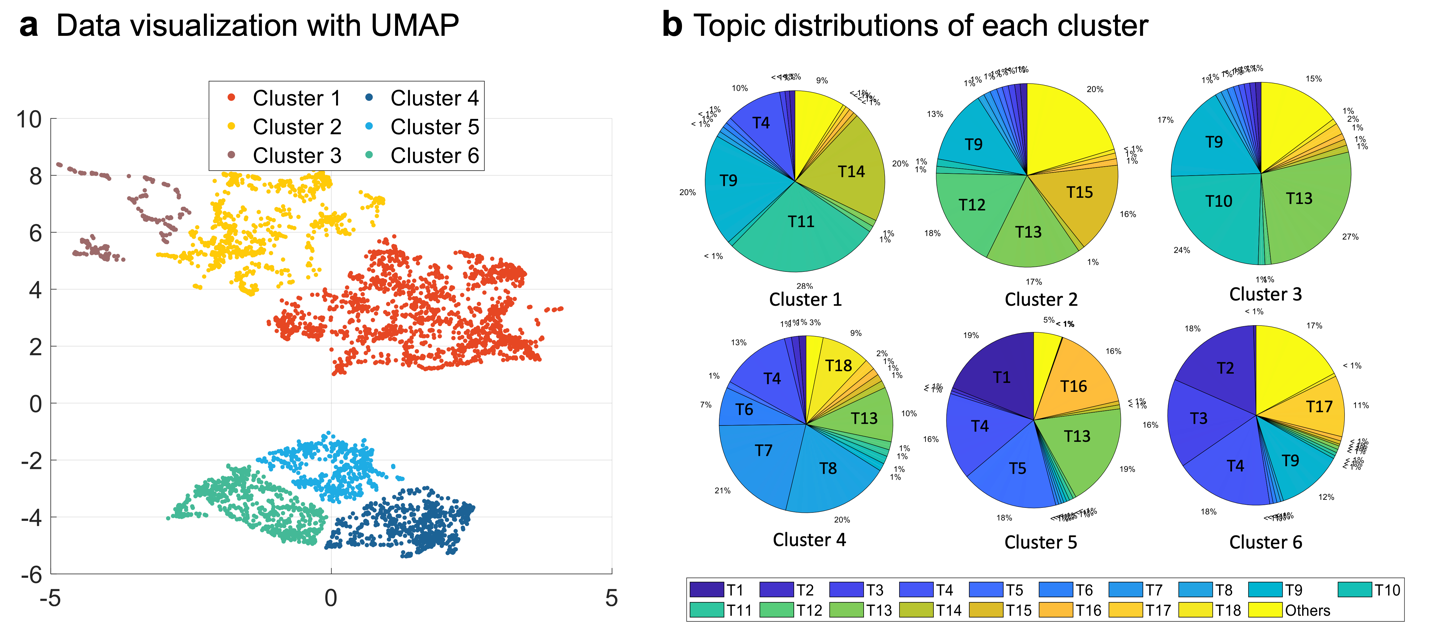


**Figure S2. Clustering results of the AD target population. a.** Data visualization with UMAP. **b.** Topic distributions of each cluster.


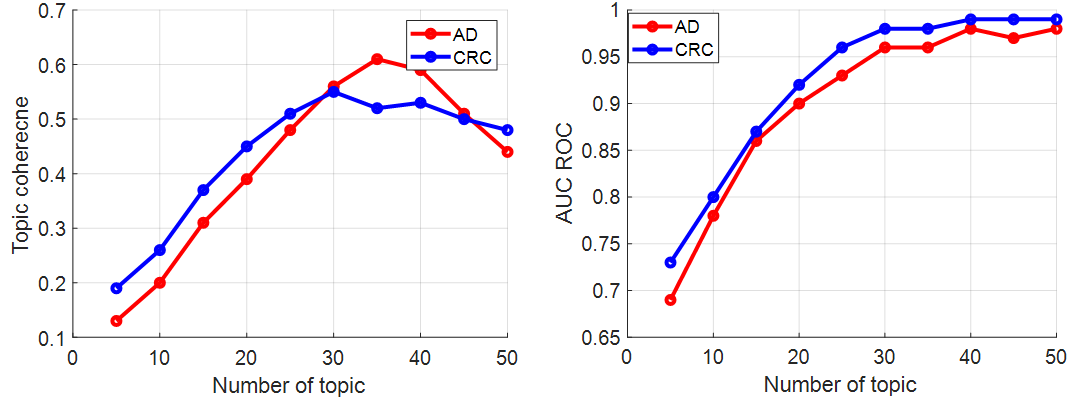


**Figure S3. The variations of topic coherence and classified AUC-ROC with the number of topics.** With the appropriate number of topics, we hope the model can achieve largest AUC-ROC (separate SAE subgroup (#SAE>0) and non-SAE subgroup (#SAE=0) well) with large topic coherence (good quality of learned topics). Therefore, we choose the number of topics as 40 for both datasets.

## Figure S4. Top features from certain disease topics (AD), which is the supplement for Figure 4c in the main manuscript.

## Figure S5. Top features from certain disease topics (CRC), which is the supplement for Figure 5c.

## Table S1. On AD dataset, for each topic, we calculate the Mean topic weight (MTW) over all samples, and p-value of Mann–Whitney U test between SAE subgroup (#SAE>0) and non-SAE subgroup (#SAE=0). We use these two values to choose typical topics.

| **Topic index** | **MTW** | **p-value** | **Topic index** | **MTW** | **p-value** | **Topic index** | **MTW** | **p-value** | **Topic index** | **MTW** | **p-value** |
| --- | --- | --- | --- | --- | --- | --- | --- | --- | --- | --- | --- |
| T1 | 0.1780 | 0.0041 | T11 | 0.1110 | 0.0021 | T21 | 0.0556 | 0.3568 | T31 | 0.0546 | 0.1082 |
| T2 | 0.1374 | 0.0092 | T12 | 0.1156 | 0.0032 | T22 | 0.0700 | 0.0426 | T32 | 0.0543 | 0.2356 |
| T3 | 0.1231 | 0.0061 | T13 | 0.2250 | 0.1657 | T23 | 0.0850 | 0.0387 | T33 | 0.0650 | 0.2903 |
| T4 | 0.2350 | 0.0753 | T14 | 0.1047 | 0.0019 | T24 | 0.0700 | 0.0197 | T34 | 0.1100 | 0.1987 |
| T5 | 0.1780 | 0.0085 | T15 | 0.1150 | 0.0067 | T25 | 0.0394 | 0.0245 | T35 | 0.1200 | 0.2354 |
| T6 | 0.1383 | 0.0026 | T16 | 0.1014 | 0.0074 | T26 | 0.0461 | 0.0324 | T36 | 0.0007 | 0.1453 |
| T7 | 0.1109 | 0.0015 | T17 | 0.1029 | 0.0048 | T27 | 0.0582 | 0.1387 | T37 | 0.0005 | 0.0664 |
| T8 | 0.1137 | 0.0033 | T18 | 0.1016 | 0.0083 | T28 | 0.0950 | 0.1957 | T38 | 0.0006 | 0.0349 |
| T9 | 0.1900 | 0.1052 | T19 | 0.0516 | 0.0264 | T29 | 0.1100 | 0.1478 | T39 | 0.0007 | 0.0279 |
| T10 | 0.1170 | 0.0089 | T20 | 0.0403 | 0.3120 | T30 | 0.0455 | 0.1754 | T40 | 0.0004 | 0.0667 |

## Table S2. On CRC dataset, for each topic, we calculate the Mean topic weight (MTW) over all samples, and p-value of Mann–Whitney U test between SAE subgroup (#SAE>0) and non-SAE subgroup (#SAE=0). We use these two values to choose typical topics.

| **Topic index** | **MTW** | **p-value** | **Topic index** | **MTW** | **p-value** | **Topic index** | **MTW** | **p-value** | **Topic index** | **MTW** | **p-value** |
| --- | --- | --- | --- | --- | --- | --- | --- | --- | --- | --- | --- |
| T1 | 0.1731 | 0.0026 | T11 | 0.1115 | 0.4310 | T21 | 0.0298 | 0.2139 | T31 | 0.0007 | 0.0275 |
| T2 | 0.1280 | 0.0038 | T12 | 0.1195 | 0.0037 | T22 | 0.0105 | 0.0917 | T32 | 0.0004 | 0.2871 |
| T3 | 0.1457 | 0.0013 | T13 | 0.1087 | 0.0021 | T23 | 0.0145 | 0.4765 | T33 | 0.0005 | 0.0015 |
| T4 | 0.1139 | 0.0006 | T14 | 0.0275 | 0.0038 | T24 | 0.0310 | 0.3681 | T34 | 0.0001 | 0.2761 |
| T5 | 0.1459 | 0.0020 | T15 | 0.0293 | 0.0632 | T25 | 0.0232 | 0.2573 | T35 | 0.0008 | 0.0983 |
| T6 | 0.1062 | 0.0042 | T16 | 0.0336 | 0. 1702 | T26 | 0.0420 | 0.7825 | T36 | 0.0002 | 0.3781 |
| T7 | 0.1672 | 0.0059 | T17 | 0.0154 | 0.0638 | T27 | 0.0005 | 0.0215 | T37 | 0.0013 | 0.0530 |
| T8 | 0.1388 | 0.0119 | T18 | 0.0280 | 0.0918 | T28 | 0.0008 | 0.3176 | T38 | 0.0010 | 0.2810 |
| T9 | 0.1283 | 0.0093 | T19 | 0.0200 | 0.1276 | T29 | 0.0003 | 0.2794 | T39 | 0.0004 | 0.0432 |
| T10 | 0.1187 | 0.2476 | T20 | 0.0275 | 0.3692 | T30 | 0.0006 | 0.3071 | T40 | 0.0021 | 0.7297 |
